# *Chlamydia *in canine or feline coronary arteriosclerotic lesions

**DOI:** 10.1186/1756-0500-4-350

**Published:** 2011-09-09

**Authors:** Ivan C Sostaric-Zuckermann, Nicole Borel, Carmen Kaiser, Zeljko Grabarevic, Andreas Pospischil

**Affiliations:** 1 Department of Veterinary Pathology, Veterinary Faculty, University of Zagreb, Heinzelova 55, 10000 Zagreb, Croatia; 2 Institute for Veterinary Pathology, University of Zurich, Vetsuisse Faculty, Winterthurerstrasse 268, CH-8057 Zurich, Switzerland

## Abstract

**Background:**

There are numerous reports linking *Chlamydia *infection to human coronary atherosclerosis. However, there is a lack of data regarding this correlation in dogs and cats, and there are no reports investigating coronary arteriosclerosis and *Chlamydia *in these species. The aim of the present study was to examine whether there is a correlation between canine and feline spontaneous atherosclerosis or arteriosclerosis and the presence of *Chlamydia*. Archived histopathological samples of dogs (n = 16) and cats (n = 13) with findings of atherosclerosis or arteriosclerosis in heart tissue were examined for the presence of *Chlamydiaceae *using real-time PCR, ArrayTube Microarray and immunohistochemistry. Additionally, arteriosclerotic lesions of all cases were histologically classified and graded.

**Results:**

Both canine atherosclerotic cases, and all 14 canine arteriosclerotic cases were negative for *Chlamydia*. Only one of the 13 arteriosclerotic feline cases was positive for *Chlamydia *by real-time PCR, revealing *C. abortus *by ArrayTube Microarray. To our knowledge, this is the first description of *C. abortus *in a cat. Overall, the type and grade of canine and feline arteriosclerotic lesions revealed similarities, and were predominantly moderate and hyperplastic.

**Conclusions:**

These findings suggest that there is no obvious correlation between canine and feline coronary arteriosclerosis and the presence of *Chlamydia*. In order to draw final conclusions about the correlation between *Chlamydia *and canine atherosclerosis, examination of more samples is required.

## Background

To date, cardiovascular disease [1] is the most prevalent cause of human mortality. Hardening of the vessels, generally denominated as "arteriosclerosis", represents one of the dominant pathological lesions involved. Most commonly, the lesion causing this hardening of the vessel walls is a formation of atherosclerotic plaques. This represents an accumulation of large amounts of lipoproteins, predominantly low density lipoproteins (LDLs) in the arterial wall, commonly called atherosclerosis. The etiology and pathogenesis of atherosclerosis seems to be quite complex and is still not completely understood. However, it was hypothesized that the initial lesion is an immunologically mediated inflammatory response by the host, presumably triggered or facilitated by certain infective agents [2]. The most extensively investigated infective agents linked with atherosclerosis include: *Helicobacter pylori*, herpesviruses and *Chlamydia *(*C*.) *pneumoniae *[2-5]. Association of *C. pneumoniae *infection and atherosclerosis has been established in many reports using a variety of methods, primarily serology, though detection of the organism in vascular lesions is also determined by isolation in culture, polymerase chain reaction (PCR), electron microscopy, enzyme immunoassay, immunohistochemistry, immunocytochemistry, Tissue Microarray and in situ hybridization [6-13]. Nevertheless, there is lack of concordance when comparing methods [14].

Incidence of atherosclerosis in dogs and cats is far less common than in humans, and is usually a sequela of other diseases, namely hypothyroidism, diabetes mellitus, hypercholesterolemia or hypertriglyceridemia [15]. However, it is still unclear whether atheromatous and/or any arteriosclerotic lesions in dogs and cats could be influenced by infectious pathogens, as described in humans. There is only one report associating chlamydial infection and atherosclerotic lesions in dogs, but none for cats [16]. There are no reports linking arteriosclerosis as a more widespread and general lesion with chlamydial infection. Dogs and cats, as common pets, usually reach a relatively high age and share the same living environment with humans. Equally importantly, they are also potential hosts for chlamydial species. Therefore it would seem logical to consider the possibility of chlamydial involvement in these vascular lesions.

The purpose of this study was to try to establish a correlation between any type of arteriosclerotic changes in hearts of dogs and cats and chlamydial infection. It is worth mentioning however, that the initial aim of this study was to try to establish a correlation between atherosclerotic lesions and chlamydial infection. Unfortunately, since the number of cases with atherosclerotic lesions was very low (n = 2), an archive search and study of cases pertaining to arteriosclerotic lesions was conducted.

## Methods

### Number and selection of cases

A search of the archives of the Institute of Veterinary Pathology in Zurich was conducted. A computer database was used to find all dog and cat postmortem cases dating from 1987 until mid-September 2010 having any description of arteriosclerotic and/or atherosclerotic lesions in heart tissues. Of the total 7,057 dog and 8,601 cat necropsies, there were 16 canine (0.23%) and 13 (0.15%) feline cases with a previous description of arteriosclerosis or atherosclerosis in heart tissue. The mean age of selected dogs and cats was 10.44 and 9.23 years, respectively. Of these 16 canine and 13 feline cases, only paraffin blocks whose hematoxylin and eosin (HE) slides were described to have some arteriosclerotic or atherosclerotic lesion were selected for further investigation.

### Preparation of HE slides and microscopic examination

For each selected paraffin block, a corresponding HE stained slide was prepared according to routine standard methods. A histopathological examination of all microscopic slides was conducted, and a grade (score) given of the arteriosclerotic/atherosclerotic lesions for each heart or aorta. Lesions of coronary arteries in heart tissues were classified into one of the five following grades: mild, moderate, severe or a combination of contiguous - e.g. mild to moderate and moderate to severe. Furthermore, using the general categorization and description of vessel wall hardening lesions, these were described for each organ as being of atherosclerotic, hyaline (also described as hypertrophic hyalinization or hyalinosis) or hyperplastic arteriosclerosis type [17,18]. Finally, the arterial and/or arteriolar lesions in each heart were examined for the presence of the following changes: visible lumen narrowing, vessel wall edema, thrombosis, and degeneration of leiomyocytes. In the determination of the previous, only hearts with a minimum of two arteries and/or arterioles affected with the change were regarded as positive findings.

### DNA extraction

Sections (40 μm) of formalin-fixed and paraffin-embedded tissue samples were deparaffinized in xylene, centrifuged at 13,500 × *g *for 5 min and the xylene was removed by repeated extraction with ethanol followed by a second centrifugation and the removal of ethanol. The pellet was treated overnight with proteinase K on a thermomixer (55°C, 550 rpm). The DNA was extracted using the DNeasy Blood and Tissue Kit (Qiagen, Hilden, Germany) according to the manufacturer's instructions.

### *Chlamydiaceae *real-time PCR

The extracted DNA of all organ samples (n = 45) was investigated on an ABI 7500 Fast real-time PCR system (Applied Biosystems, Foster City, CA, USA) using the 23S-based *Chlamydiaceae *family-specific real-time PCR described previously [19]. This method includes primers Ch23S-F (5'-CTGAAACCAGTAGCTTATAAG CGGT-3'), Ch23S-R (5'-ACCTCGCCGTTTAACTTAACTCC-3'), and probe Ch23S-p (FAM-CTCATCA TGCAAAAGGCACGCCG-TAMRA) and an internal amplification control consisting of primers EGFP-1-F (5'-GACCACTACCAGCAGAACAC-3'), EGFP-10-R (3'-CTTGTACAGCTCGTCCATGC-5') and probe EGFP-HEX (HEX-AGCACCCAGTCCGCCCTGAGCA-BHQ1). A 111-bp product specific for members of the *Chlamydiaceae *is also produced as well as a 177-bp product for the internal amplification control. A final volume of 25 μl for each tested sample was achieved by adding 2.5 μl of extracted DNA to 22.5 μl of 2X TaqMan^® ^Fast Universal PCR Master Mix (Applied Biosystems) and a final concentration of 500 nM of each primer and the probe (Microsynth, Balgach, Switzerland). The cycling program was started by an initial denaturation (95°C, 20 sec) followed by 45 cycles of denaturation and annealing (95°C, 3 sec and 60°C, 30 sec), with an automatically calculated cycle threshold value. All samples were tested in duplicate. If the duplicates showed a Ct value of < 38, the sample was considered positive and if the mean Ct value was > 38, it was considered questionably positive.

### ArrayTube (AT) Microarray for species identification of *Chlamydiaceae*

Samples that were positive or considered questionably positive by real-time PCR for *Chlamydiaceae *were further examined by the species-specific 23S ArrayTube (AT) Microarray [20].

### Immunohistochemistry (IHC)

The presence of the chlamydial antigen in paraffin sections was investigated on cases positive or considered questionably positive for *Chlamydiaceae *by real-time PCR (n = 1) using a *Chlamydiaceae *family-specific mouse monoclonal antibody directed against the chlamydial lipopolysaccharide (LPS, Clone ACI-P, Progen, Heidelberg, Germany). A detection kit (Dako, K5003, Switzerland) was used according to the manufacturer's instructions for detection. After deparaffinization in xylene and rehydration through graded ethanol to water, the antigen retrieval was performed by 10 min enzyme digestion (Proteinase K, Dako, S2019, Switzerland). Endogenous peroxidase activity was inhibited with peroxidase-blocking solution for 5 min at room temperature, then slides were incubated for 60 min with the primary antibody diluted 1:200 in antibody diluent (S2022, Dako, Switzerland). The incubation with the link-antibody and the substrate solution was performed at RT for 10 min each, after which the slides were developed in a 3-amino, 9-ethyl-carbazole (AEC) substrate solution for 10 min and counterstained with hematoxylin. Negative controls of each section were performed using only the antibody diluent instead of the primary antibody. For the positive control, intestinal tissue from gnotobiotic piglets experimentally infected with porcine *C. suis *strain S45 were used [21].

## Results

### Number and selection of cases

Of dogs, 15 animals (93.8%) had recorded lesions only in the heart (n = 14) or aorta (n = 1), while one animal (6.2%) showed lesions in the heart and some additional organs (kidney and lungs). Of cats, seven animals (53.8%) expressed arteriosclerotic lesions only in the heart, while the remaining six animals (46.2%) had both coronary and non-coronary lesions (kidney, eyes, liver, brain, spleen, stomach).

### Microscopic evaluation and grading of the lesions

#### Dogs

**Figure 1:**
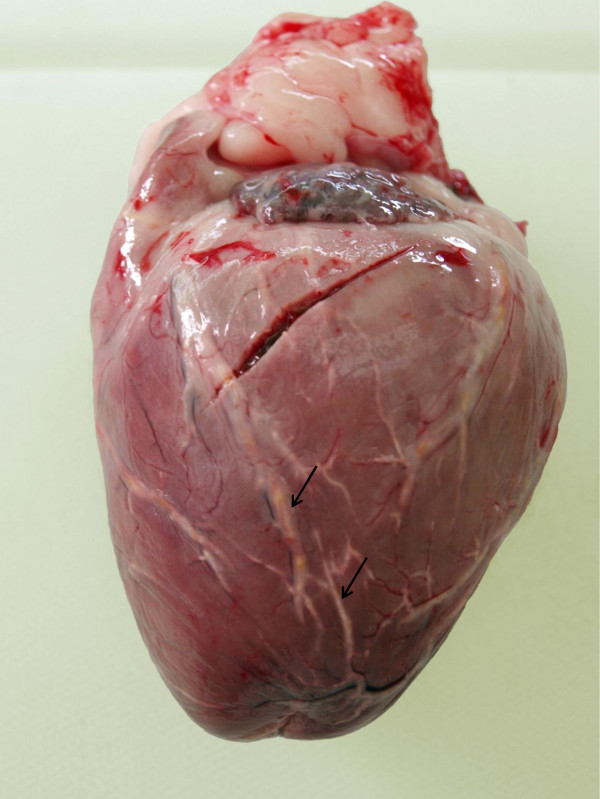
**Heart, dog, case No. 2**. Macroscopic view of the left ventricle of the heart. Coronary arteries with pronounced atherosclerotic changes are clearly visible as white, broadened, slightly bulging streaks (arrows). Oblique cut toward the base of the heart is an artefact.

**Figure 2:**
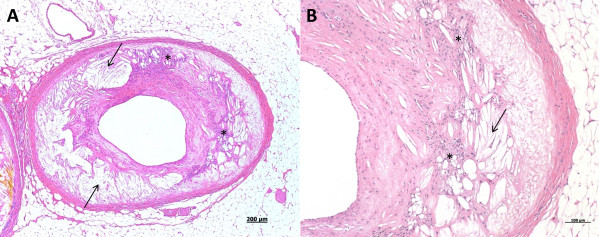
**Heart, dog, case No. 2**. Two micrographs depicting the same, severe atherosclerotic lesion of a coronary artery (**2a - **lower magnification, **2b - **higher magnification). A major section of the arterial wall has been replaced and distorted by acicular cholesterol clefts (arrows). Inflammatory cells, mostly consisting of lipid laden histiocytes (asterisk) are visible within the arterial wall.

**Figure 3:**
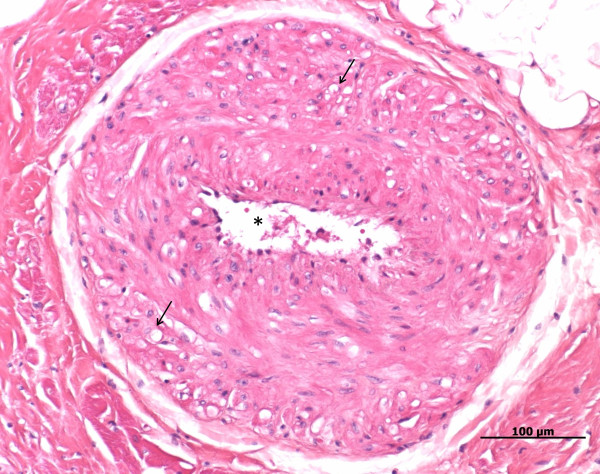
**Heart, dog, case No. 9**. Hyperplastic arteriosclerosis of a branch of coronary artery. Note the thickened vessel wall and extremely narrowed lumen (asterisk). In addition to being hyperplastic, some leiomyocytes of the vessel wall are also degenerated, seen by the clear vacuoles in the cytoplasm (arrows).

**Figure 4:**
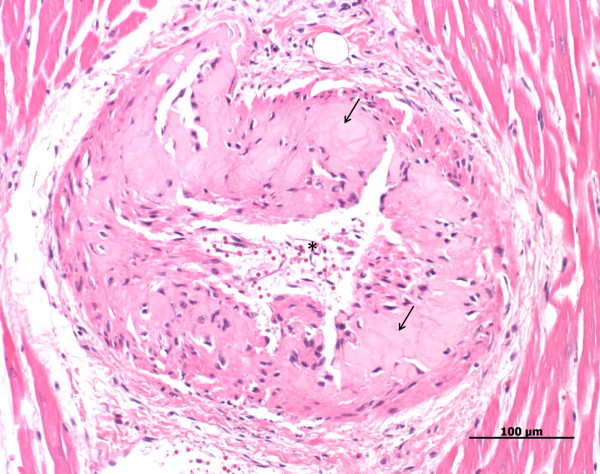
**Heart, dog, case No. 15**. Hyaline arteriosclerosis of a branch of the coronary artery. Note the irregular and narrowed lumen of the artery (asterisk). Acellular areas with an accumulation of eosinophilic, lightly translucent, protein like (hyaline) material (arrows) are visible in the vessel wall.

Details of the 16 dogs are shown in Table 1. Only two cases had atherosclerotic lesions, while the remaining lesions were classified as arteriosclerosis (n = 14). Macroscopic and microscopic examples of atherosclerotic changes in the coronary arteries of the heart of dog no 2 are shown in Figure 1 and 2a, respectively. Higher magnification of the atherosclerotic lesion of case no. 2 is shown in Figure 2b. The most common type of arteriosclerotic lesions in the coronary arteries was hyperplastic (n = 12, Figure 3), occurring either alone (n = 7/12) or in combination with hyaline arteriosclerosis (n = 5/7, Figure 4). The grade of all lesions (n = 16) varied from mild to severe in all possible combinations, with moderate to severe as the most common (31.3%). Lumen narrowing was present in 15 cases (93.8%), followed by thrombosis in seven cases (43.8%), vessel wall edema (n = 3, 18.8%), and degeneration of leiomyocytes (n = 2, 12.5%).

**Table 1:** Type and grade of atherosclerotic and arteriosclerotic lesions in hearts and aorta of dogs (n = 16)

No	Breed	Sex	Age	Organ	Grade	Type	Other organs affected	Concurrent lesions in the heart and other organs
1	Mongrel	M	11	Heart	++ to +++	atherosclerosis	none	DIC lung, chronic cholecystitis, necrosis liver & spleen

2	Mongrel	M	13	Heart	+++	atherosclerosis	none	Chemodectoma heart

3	Bull terrier	F	11	Heart	+	hyperplastic	none	Amyloidosis kidney, liver, spleen, heart & intestine

4	Neapolitan Mastiff	MC	4	Heart	+ to ++	hyperplastic	none	Glomerulonephritis, heart infarct

5	Dachshund	M	17	Heart	+ to ++	hyperplastic	none	Endocardiosis AV valves

6	St. Bernard	M	4	Heart	++	hyperplastic	none	Dilatative cardio-myopathy

7	Mongrel	MC	11	Heart	++	hyperplastic	lung, kidney	Chronic lung hyper-tension, chronic interstitial nephritis

8	Scottish terrier	F	12	Heart	++ to +++	hyperplastic	none	Hepatoma liver

9	Golden Retriever	FC	12	Heart	++ to +++	hyperplastic	none	Intestinal lymphoma

10	Bernese mountain dog	F	2	Heart	+ to ++	hyperplastic and hyaline	none	Chronic glomerulo-nephritis & interstitial nephritis

11	Large Münsterländer	F	13	Heart	++	hyperplastic and hyaline	none	Hemangiosarcoma heart & kidney

12	Jack Russel terrier	F	11	Heart	++ to +++	hyperplastic and hyaline	none	Myocardial fibrosis & AV endocardiosis left

13	German shepherd	M	13	Heart	+++	hyperplastic and hyaline	none	endocardiosis AV left & Aortic valves

14	Bernese mountain dog	M	9	Heart	+++	hyperplastic and hyaline	none	Hemangiosarcoma kidney

15	Dobermann	F	11	Heart	++ to +++	hyaline	none	Chronic interstitial nephritis

16	Manchester terrier	FC	13	Aorta	++	hyaline	none	Glomerulonephritis

+ mild; ++ moderate; +++ severe; - not present

AV, Atrioventricular; M, Male; F, Female; MC, Male Castrated, FC, Female Castrated;

#### Cats

Arteriosclerosis was present in all 13 cat hearts, as evident in Table 2. None of these cases showed atherosclerotic lesions. The most frequent type of arteriosclerotic lesions was hyperplastic arteriosclerosis, occurring either alone (n = 9, 69.2%) or in combination with hyaline arteriosclerosis (n = 11, 84.6%). The grade of all lesions again varied from mild to severe in all possible combinations, with moderate as the most common (53.8%). Lumen narrowing was observed in 12 cases (92.3%), followed by degeneration of leiomyocytes and vessel wall edema in four (30.8%), and thrombosis in three cases (23.1%).

**Table 2:** Type and grade of arteriosclerotic lesions in hearts of cats (n = 13)

No	Breed	Sex	Age	Organ	Grade	Type	Other organs affected	Concurrent lesions in the heart or other organs
1	Persian cat	MC	8	Heart	+	hyperplastic	none	Dilatative cardio-myopathy, chronic interstitial nephritis

2	European shorthair	FC	8	Heart	+ to ++	hyperplastic	none	Chronic interstitial nephritis

3	European shorthair	FC	?	Heart	+ to ++	hyperplastic	liver, kidney, spleen	Chronic interstitial nephritis, hypertensive retinopathy

4	European shorthair	MC	12	Heart	++	hyperplastic	none	Chronic interstitial nephritis, heart infarcts

5	European shorthair	F	?	Heart	++	hyperplastic	none	Fibrosis of the atrio-ventricular node

6	European shorthair	F	15	Heart	++	hyperplastic	kidney, eye, brain	Hypertrophic cardio-myopathy, glomerulo-nephritis

7	British shorthair	MC	5	Heart	++	hyperplastic	none	Left ventricular hyper-trophy, myocardial fibrosis

8	European shorthair	FC	15	Heart	++	hyperplastic	eye	Thyroid adenoma, hypertrophic cardio-myopathy, chronic interstitial nephritis

9	European shorthair	FC	6	Heart	++ to +++	hyperplastic	none	Chylothorax, myocardial fibrosis & necrosis

10	European shorthair	F	13	Heart	++	hyperplastic and hyaline	spleen, kidney, stomach, heart, meninges, eyes	Chronic interstitial nephritis

11	European shorthair	M	1	Heart	+++	hyperplastic and hyaline	kidney	Hypertrophic cardio-myopathy, chronic interstitial nephritis

12	Burmese cat	MC	16	Heart	++	hyaline	brain	Hypertrophic cardio-myopathy, chronic interstitial nephritis

13	Birman	FC	1.5	Heart	+++	hyaline	none	Brain edema

+ mild; ++ moderate; +++ severe; - not present

M, Male; F, Female; MC, Male Castrated, FC, Female Castrated;

### *Chlamydiaceae *real-time PCR

Of all the samples (hearts and other organs affected in these animals, n = 45), only one was considered questionably positive by real-time PCR (with a Ct value of 38.5). This questionably positive sample was from a cat heart (case no. 3).

### ArrayTube (AT) Microarray for species identification of *Chlamydiaceae*

The only questionably positive sample by real-time PCR (cat case no. 3) was investigated by the species-specific 23S AT Microarray, and the presence of *C. abortus *specific DNA was detected in the sample.

### Immunohistochemistry (IHC)

No positive labelling was observed in the feline heart tissue of the only *Chlamydiaceae *real-time PCR questionable case positive by AT Microarray for *C. abortus *(cat case no. 3).

## Discussion

The aim of this study was to correlate spontaneous atherosclerotic and arteriosclerotic lesions in heart tissues of dogs and cats with the presence of *Chlamydia*. Regarding solely atherosclerotic cases, no connection was established between these lesions and chlamydial infections. However, it was not possible to draw final conclusions from these results, since there were only two cases showing atherosclerotic changes. These findings are contrary to those from a similar study [16] in which the authors found the presence of *C. psittaci *and *C. pneumoniae *in all seven canine atherosclerotic cases investigated in the study by using immunohistochemical methods. They subsequently confirmed their findings by conducting a *C. pneumoniae*-specific PCR in one of these cases, which yielded a positive result. They also identified *Chlamydia *in an unspecified number of cases using electron microscopy. In the present study, a different diagnostic approach was chosen. As it has been postulated that immunohistochemistry can yield false positive results due to a cross-reaction of chlamydial antibodies with microcalcifications and insoluble lipid ceroids, a two-step approach was applied here with initial screening by real-time PCR for *Chlamydiaceae *followed by the ArrayTube Microarray and immunohistochemistry [14]. This combination of methodologies was advised for the identification of *Chlamydia *in animal tissue samples in a recent review paper [22]. The different diagnostic approach and the low number of atherosclerosis cases could explain the different results when comparing this study with the study by Sako *et al*., 2002 [16]. Considering the latter, it would be highly advisable in any future studies that a larger number of atherosclerotic cases be examined.

On account of the very low number of coronary atherosclerosis cases in dogs and cats yielded by the database search, coronary arteriosclerotic cases were included in this study. To date, there are no reports in veterinary medicine linking arteriosclerosis with the presence of *Chlamydia*. However, in human medicine there is a publication indicating an association between the presence of *C. pneumoniae *and early calcification of the tunica media in arteriosclerotic coronary arteries [23]. Thus, it seemed worthwhile to investigate coronary arteriosclerotic lesions from dogs and cats. In conclusion, examination of arteriosclerotic lesions in 14 dogs and 13 cats, did not reveal any presence of *Chlamydia *in dogs, and found only one positive case in a cat. This low incidence of positive cases indicated that chlamydial infections are not directly linked to the development of canine and feline arteriosclerotic lesions. In general, reports on chlamydial infections in dogs of Switzerland are scarce, and are either case reports of *C. abortus *induced keratoconjunctivitis, or studies that failed to prove the presence of *Chlamydia *in dogs with or without idiopathic pericardial effusion [24,25]. This lack of data could either be due to a low prevalence of canine chlamydial infections in this country or due to limited investigations on this animal species. Considering the data from other countries, Holst *et al*., 2010 found no *Chlamydia *from the ocular or genital swabs of healthy Swedish dogs or those showing genital or ocular clinical signs, indicating either a low prevalence or absence of chlamydial infections in these animals [26]. However, there are previous reports of *Chlamydia-*associated disease in dogs. *C. psittaci *in dogs can cause respiratory disease, conjunctivitis and listlessness [27,28].

When comparing the situation in cats, it is well known that *Chlamydia *is considered a cause of ocular disease in cats worldwide, including Switzerland [29,30]. Therefore Chlamydia is more likely to be found in association with cats than it is with dogs. The present results, however, do not point in this direction, since only one *Chlamydia*-positive case was found in coronary arteriosclerotic samples from cats. In older studies, *C. psittaci *(old classification) was implicated as an etiologic agent of pneumonia in cats [31,32]. As such, a parallel with the development of human atherosclerosis, where *C. pneumoniae *is thought to travel from the patient's lung to coronary arteries (proven in a murine model), could have been proposed [33]. However, these speculations are no longer relevant for the pathogenesis of atherosclerosis and *Chlamydia *in cats as newer reports indicate that *Chlamydia *is not involved in feline pneumonia [34].

The chlamydial species in the only positive feline case was neither *C. pneumoniae*, the most common chlamydial species involved in human cardiovascular lesions, nor *C. felis*, a recognized pathogen of feline conjunctivitis, but instead was *C. abortus *[29,35]. This chlamydial species is known worldwide as a cause of abortions in ruminants - e.g. *ovine enzootic abortion *in sheep, but has not previously been described in cats [36]. The occurrence of a particular chlamydial species in another host was already implicated in the work of Pantchev *et al*., 2010, showing that the host range of a particular chlamydial species is not as strict as previously thought [35]. Thus, more research should be conducted in identifying the possible range of chlamydial species in various chlamydial diseases and different hosts.

The very low number of atherosclerotic cases recorded during more than 20 years of necropsies (7,057 necropsies of dogs, and 8,601 necropsies of cats) showed the low incidence of this condition in dogs (n = 2; 0.028%), and absence of the condition in cats (n = 0). Likewise, the total number of arteriosclerotic lesions in dogs (n = 14; 0.2%) and cats (n = 13; 0.15%) was quite low for such a long period, which could imply that arteriosclerosis does not play a very important role in the development of heart pathologic conditions in these species. The literature search did not yield any reports on the incidence of canine or feline coronary arteriosclerosis that would support the present findings. According to textbooks, arteriosclerosis is quite common in older dogs and cats and it has the potential to cause coronary ischemia, but not very often [18].

The present results on the histopathological classification of arteriosclerotic lesions showed similarities between the distribution of lesions according to arteriosclerosis grade and type between dogs and cats. However, 46.2% of cats were found to have simultaneous coronary and extracoronary lesions, as compared to only 6.2% of such cases in dogs. Thus, arteriosclerosis seems to be a more systemic disease in cats, and is not strictly associated with the heart. One explanation for this could be that in many selected feline cases, interstitial nephritis was also present (data not shown). Thus, severe interstitial nephritis may have caused end stage kidney and renal failure, which is known to cause hypertension that in turn leads to arteriosclerotic changes first in renal arteries and then systemic [18].

## Conclusions

In conclusion, this investigation indicates that there is no obvious correlation between spontaneous coronary arteriosclerotic lesions of dogs and cats and chlamydial infections. Regarding the association of *Chlamydia *and atherosclerosis, more samples of canine and feline atherosclerotic lesions should be investigated to draw final conclusions on the relevance of *Chlamydia *as an inducer or bystander of such lesions.

## Declaration of competing interests

The authors declare that they have no competing interests.

## Authors' contributions

ICSZ conducted most of the laboratory work, performed the light microscopy and drafted the manuscript. NB devised the design of the study, participated in drafting of the manuscript and assisted with light microscopy. CK participated in laboratory work. ZG and AP participated in design of the study and contributed to writing the manuscript. All authors read and approved the final version of the manuscript.
